# Evaluation of multiplex polymerase chain reaction as an alternative to conventional antibiotic sensitivity test

**DOI:** 10.14202/vetworld.2018.474-479

**Published:** 2018-04-13

**Authors:** K. Rathore, B. Joseph, D. K. Sharma, A. Gaurav, S. K. Sharma, M. Milind, P. Patel, C. Prakash, L. Singh

**Affiliations:** 1 Department of Veterinary Microbiology, College of Veterinary and Animal Science, Navania, Udaipur, 313601, Rajasthan, India; 2 Department of Veterinary Public Health, College of Veterinary and Animal Science, Navania, Udaipur, 313601, Rajasthan, India; 3 Department of Veterinary Medicine, College of Veterinary and Animal Science, Navania, Udaipur, 313601, Rajasthan, India; 4 Animal Health Division, CSWRI, Avikanagar, Malpura, Tonk, 304501 Rajasthan, India; 5 Department of Livestock Products Technology, College of Veterinary and Animal Science, Navania, Udaipur, 313601, Rajasthan, India

**Keywords:** antimicrobial resistance, genotype, phenotype, *Staphylococcus aureus*

## Abstract

**Aim:**

This study was designed to evaluate the potential of the use of multiplex polymerase chain reaction (PCR) as an alternative to conventional antibiotic sensitivity test.

**Materials and Methods:**

Isolates of *Staphylococcus aureus* (total = 36) from clinical cases presented to Teaching Veterinary Clinical Complex of College of Veterinary and Animal Sciences (CVAS), Navania, Udaipur, were characterized by morphological, cultural, and biochemical methods. Then, the isolates were further subjected to molecular characterization by PCR targeting *S. aureus*-specific sequence (107 bp). Phenotypic antibiotic sensitivity pattern was analyzed by Kirby-Bauer disc diffusion method against 11 commonly used antibiotics in veterinary medicine in and around Udaipur region. The genotypic antibiotic sensitivity pattern was studied against methicillin, aminoglycosides, and tetracycline targeting the gene *mecA*, *aacA*-*aphD*, and *tetK* by multiplex PCR.

**Results:**

There was 100% correlation between the phenotype and genotype of aminoglycoside resistance, more than 90% correlation for methicillin resistance, and 58.3% in the case tetracycline resistance.

**Conclusion:**

As there is a good correlation between phenotype and genotype of antibiotic resistance, multiplex PCR can be used as an alternative to the conventional antibiotic susceptibility testing, as it can give a rapid and true prediction of antibiotic sensitivity pattern.

## Introduction

Antimicrobial resistance is becoming an extremely serious global health problem [1]. The liberal use of antimicrobials for treatment in human beings and animals as well as the subtherapeutic use in livestock for growth promotion and prophylaxis has greatly contributed to the emergence and persistence of resistant strains of bacteria [2,3]. Animal-to-human, as well as human-to-animal transmission of resistant strains, can have a potential impact on public health if these strains enter into the community and health-care settings. Methicillin-resistant *Staphylococcus aureus* (MRSA) is one of the highest ranking pathogens worldwide and represents a real challenge to the clinical practice with significant public health concern [4].

Accurate and rapid identification of MRSA and their antimicrobial susceptibility profile is essential for the selection of appropriate therapy [5]. Therefore, it is clinically crucial to determine quickly whether *S. aureus* isolates are methicillin-resistant or not, as this is very significant for both treatment and limit the spread of such strains. Early diagnosis and appropriate treatment can significantly limit the duration and outcome of infection. Rapid molecular diagnostic platforms are useful to clinicians when the prevalence of resistance for a given population is to be determined [6].

Multiplex polymerase chain reaction (PCR) assay for accurate and timely detection of multidrug-resistant organism and resistance markers will help the clinicians in making early antibiotic adjustments [7]. Multiplex PCR is a useful tool for multidrug-resistant *Staphylococcus* monitoring in clinical laboratories [8] which will take only 6 h to give the result compared to antibiotic sensitivity test which takes 2 days for the completion of result [9]. The multiplex PCR approach can be a beneficial adjunct to standard microbiological methods for rapid and specific identification of pathogens and resistance patterns [10]. The present study aimed to establish the importance of multiplex PCR assay as an alternative to conventional antibiotic sensitivity test.

## Materials and Methods

### Ethical approval

In this investigation, we did not use live animals. The clinical samples submitted to the Department of Microbiology, CVAS, Navania was used for isolation of bacteria and further characterization. Therefore, no ethical approval was needed for the present study.

### Isolation and identification of S. aureus

A total of 60 samples comprising milk, pus, body fluids, uterine and vaginal discharge, nasal swab, ear discharge, and postmortem samples of animals of different species including cattle, buffalo, sheep, goat, and poultry were processed for the isolation of *S. aureus*. Each clinical sample was inoculated into 5 mL of Nutrient broth (HiMedia) and incubated at 37°C overnight. Next day, it is subcultured on to Nutrient agar (HiMedia), McConkey agar (HiMedia), and Mannitol salt agar (HiMedia) and then incubated at 37°C for 24-48 h. The organism is characterized morphologically, culturally, and biochemically [11].

### Molecular characterization of S. aureus

#### Genomic DNA extraction

Extraction of genomic DNA was performed from bacterial cultural isolates following cetyltrimethylammonium bromide (CTAB) method with slight modifications [12]. Bacterial pellet was obtained by centrifugation of overnight culture of *S. aureus* at 5000 rpm for 5 min at 4°C. The pellet was resuspended in 567 µL of TE buffer by repeated pipetting. Then, 30 µL of 10% SDS and 3 µL of 20 mg/mL proteinase K were added to give a final concentration of 100 µg/mL proteinase K in 0.5% SDS, mixed thoroughly, and incubated at 37°C for 1 h. The lysate was treated with 100 µL of 5M NaCl and 80 µL CTAB (10% w/v in 0.7 M NaCl w/v) with further incubation at 65°C in a water bath for 10 min. An equal volume of phenol: chloroform:isoamyl alcohol (25:24:1 w/v) were added in each tube, mixed gently, and centrifuged at 13,000 rpm for 5 min. The supernatant was collected in a separate tube and DNA was precipitated by incubating the tubes at −20°C overnight after adding 1/10 volume of ammonium acetate (7.5 M) and double the volume of chilled absolute ethanol. Finally, the DNA pellet was obtained by centrifugation at 13,000 rpm for 20 min at 4°C. The DNA pellet was washed with 70% ethanol dried and resuspended in 100 µL sterile nuclease-free water. DNA was finally stored at −80°C in small aliquots.

### PCR amplification

Genomic DNAs extracted were used to *S. aureus*-specific sequence (107 bp) by PCR [9]. The oligonucleotide primers used for the amplification is mentioned in Table-1. PCR assay was performed in a final volume of 25 µL mixture containing 10X PCR buffer with 1.5 mM MgCl_2_ (2.5 µL), 0.2 mM of each deoxynucleotide triphosphate (0.5 µL), 1.25 unit *Taq* DNA polymerase (0.4 µL) (Fermentas), 0.5 µmol of each primer, and 1 µL DNA template. The amplification was carried out with 30 cycles and PCR conditions used are presented in Table-2. The PCR products were analyzed using 1.2% (w/v) agarose gel with 0.5 µg/mL ethidium bromide using 1X TAE electrophoresis buffer.

**Table-1:** Details of primers used in the present study.

Oligo name	Sequence 5’‑->3’	Gene	Tm (°C)	GC-content (%)	Amplicon size (bp)
*mecA* (forward)	5’AAAATCGATGGTAAAGGTTGGC3’	*mecA*	56.5	40.90	532
mecA (reverse)	5’AGTTCTGCAGTACCGGATTTGC3’		60.3	50	
*aacA-aphD* (forward)	5’TAATCCAAGAGCAATAAGGGC3’	*aacA-aphD*	55.9	42.90	227
*aacA-aphD* (reverse)	5’GCCACACTATCATAACCACTA3’		55.9	42.90	
*tetK* (forward)	5’GTAGCGACAATAGGTAATAGT3’	*tetK*	54	38.10	360
*tetK* (reverse)	5’TAATCGTGGAATACGGGTTTG3’		55.9	42.90	
16s 1 (forward)	5’CAGCTCGTGTCGTGAGATGT3’	16s rRNA	59.4	55	420
16s 2 (reverse)	5’AATCATTTGTCCCACCTTCG3’		55.3	45	
*sau* 1 (forward)	5’AATCTTTGTCGGTACACGATATTCTTCACG3’	*S. aureus* specific sequences	64	40	107
*sau* 2 (reverse)	5’CGTAATGAGATTTCAGTAGATAATACAACA3’		59.9	30	

S. aureus=Staphylococcus aureus

**Table-2:** PCR conditions for *S. aureus*-specific sequencesbased characterization.

Gene (size)	PCR conditions		

	Denaturation	Annealing	Extension
*S. aureus* specific sequences (107 bp)	94°C for 1 min	55°C for 1 min	72°C for 1 min

*S. aureus=Staphylococcus aureus*, PCR=Polymerase chain reaction

### Phenotypic antibiotic sensitivity test pattern of S. aureus isolates

The disc diffusion method [13] was followed to determine the antibiogram of the isolates against 11 commonly used antibiotics; amoxyclav (30 mcg), ceftriaxone (10 mcg), ciprofloxacin (5 mcg), methicillin (5 mcg), ampicillin (10 mcg), chloramphenicol (30 mcg), tetracycline (30 mcg), gentamicin (10 mcg), streptomycin (10 mcg), norfloxacin (10 mcg), and amikacin (30 mcg) in veterinary medicine in and around Udaipur district of Rajasthan.

### Genotypic antibiotic sensitivity pattern of S. aureus isolates by multiplex PCR

Genotypic characterization of antibiotic sensitivity pattern against methicillin, aminoglycoside, and tetracycline was done by amplification of *mecA*, *aacA*-*aphD*, and *tetK* gene by multiplex PCR [9]. In this study previously published, primers and PCR reaction conditions are used [9]. The details of the primers used in the study are shown in Table-1.

Multiplex PCR amplifications were carried out in a 100 µL volume comprising approximately 40 ng of template DNA, 10 pmol of each of the six primers, a final concentration of 0.4 mM each deoxyribonucleoside triphosphate, and 5U of *Taq* DNA polymerase (Fermentas) in 1× PCR buffer supplied by manufacturer; final concentration of MgCl_2_ in the PCR mixture was adjusted to 4 mM. The following cycling conditions were used in the multiplex PCR: Initial denaturation at 94°C for 3 min was followed by 30 cycles of amplification with 94°C for 30 s, annealing at 55°C for 30 s, and extension at 72°C for 30 s and a final extension of 72°C for 4 min. The PCR products were analyzed on a 2.5% agarose gel to separate different amplification products efficiently.

## Results

### Isolation, identification, and biochemical characterization of S. aureus isolates

Out of 60 processed clinical samples, 36 isolates showing typical morphological, biochemical, and cultural characteristics of *S. aureus* were obtained.

### Molecular characterization

**Figure-1:**
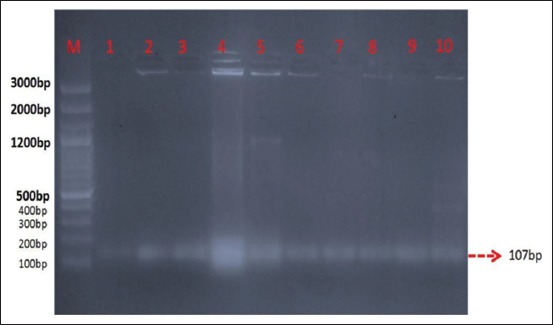
Polymerase chain reaction amplification of *Staphylococcus aureus*-specific sequence (107 bp).

All the 36 isolates of *S. aureus* were subsequently characterized by molecular methods. All the genomic DNA preparations of 36 isolates of *S. aureus* showed an amplified product 107 *bp* for *S. aureus*-specific sequences (Figure-1).

### Phenotypic antibiotic sensitivity pattern of S. aureus

Phenotypic antibiotic resistance pattern of *S. aureus* is depicted in Table-3. It was observed that MRSA was also resistant to other antibiotics compared to methicillin-susceptible *S. aureus* (MSSA) which were found susceptible to other antibiotics also.

**Table-3:** Phenotypic antibiotic resistance pattern of *S. aureus* isolates from various clinical samples using 11 commonly used antibiotics in veterinary field conditions by Kirby–Bauer disc diffusion method.

*S. aureus* isolate number	Phenotypic antibiotic sensitivity pattern
373	AMC, CTR, CIP, MET, AMP, TET, GEN, S, Nx, AK
413	MET, AMP, C
415	AMC, CTR, CIP, TET, GEN, S, Nx, AK
417	AMC
418	MET, TET
419	AMC, CTR, CIP, MET, TET, GEN, S, Nx, AK
423	AMC, CTR, MET, TET, S
424	AMC, CTR, CIP, MET, TET, GEN, S, Nx, AK
427	AMC, CTR, CIP, MET, AMP, TET, GEN, S, Nx, AK
430	MET, AMP
431	AMP
432	CTR, CIP, MET, AMP, C, TET, GEN, S
433	CTR, MET, AMP
496	AMC, CTR, CIP, MET, AMP, C, TET, S
502	AMC, CTR, CIP, MET, AMP, TET, Nx
508	AMC, CTR, CIP, MET, C, TET, GEN, S, Nx, AK
510	AMC, MET, AMP, C
515	AMC, CTR, MET, AMP, TET, GEN, S, Nx
532	AMC, CTR, CIP, MET, AMP, C, TET, GEN, S, Nx, AK
534	AMC, CTR, CIP, MET, AMP, C, TET, GEN, S, Nx, AK
536	AMC, CTR, CIP, MET, AMP, C, TET, GEN, S, Nx, AK
537	AMC, CTR, CIP, MET, AMP, C, TET, GEN, S, Nx, AK
538	AMC, CTR, CIP, MET, AMP, C, TET, GEN, S, Nx, AK
541	AMC, CTR, CIP, MET, AMP, C, TET, GEN, S, Nx, AK
542	AMC, CTR, CIP, MET, AMP, C, TET, GEN, S, Nx, AK
543	AMC, CTR, CIP, MET, AMP, C, TET, GEN, S, Nx, AK
544	AMC, CTR, CIP, MET, AMP, C, TET, GEN, S, Nx, AK
545	CTR, CIP, MET, TET, Nx
555	AMC, CTR, MET, AMP, C, TET, S, Nx
556	AMC, CTR, CIP, MET, AMP, C, TET, GEN, S, Nx, AK
557	AMC, CTR, CIP, MET, AMP, C, TET, GEN, S, Nx, AK
558	AMC, CTR, CIP, MET, AMP, C, TET, S
559	AMC, CTR, CIP, MET, AMP, C, TET, GEN, S, Nx, AK
560	AMC, CTR, CIP, MET, AMP, C, TET, GEN, S, Nx, AK
561	AMC, CTR, CIP, MET, AMP, C, TET, GEN, S, Nx, AK
562	AMC, CTR, CIP, MET, AMP, C, TET, GEN, S, Nx, AK

*S. aureus=Staphylococcus aureus*, AMC=Amoxyclav, CTR=Ceftriazone, CIP=Ciprofoxacin, MET=Methicillin, AMP=Ampicillin, C=Chloramphenicol, TET=Tetracycline, GEN=Gentamicin, S=Streptomycin, Nx=Norfloxacin, AK=Amikacin

### Genotypic antibiotic resistance pattern of S. aureus isolates

**Figure-2:**
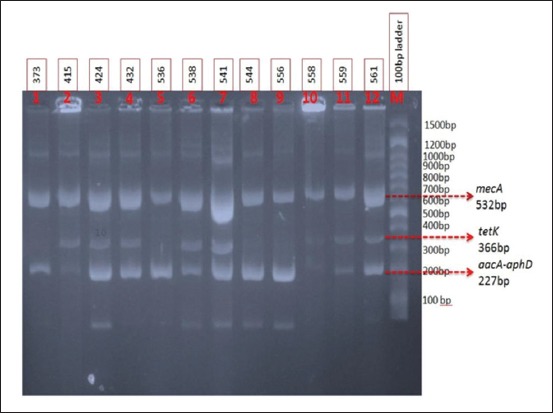
Genotypic characterization of antibiotic resistance pattern of different isolates of *Staphylococcus aureus* against methicillin, aminoglycosides, and tetracycline using multiplex polymerase chain reaction. Lane 1-12: Genotype of antibiotic resistance pattern of *S. aureus* isolated number 373, 415, 424, 432, 536, 538, 541, 544, 556, 558, 559, and 561, respectively, Lane M: 100 bp ladder.

Genotypic antibiotic resistance pattern of 12 randomly selected isolates (isolate no. 373, 415, 424, 432, 536, 538, 541, 544, 556, 558, 559, and 561) were characterized by multiplex PCR. Multiplex PCR assay was used for the detection of three clinically relevant antibiotic resistant genes of *S. aureus* such as *mecA* (encoding methicillin resistance), *aacA-aphD* (aminoglycoside resistance), and *tetK* (tetracycline resistance) simultaneously in single PCR amplification. The results of multiplex PCR were depicted in Figure-2.

### Correlation between phenotypic and genotypic antibiotic resistance pattern

For 12 *S. aureus* isolates, we compared susceptibility results determined by Kirby–Bauer disc diffusion test with the results of the multiplex PCR assay for the simultaneous detection of antibiotic-resistant genes. For these isolates, we found a correlation between the results of multiplex PCR and those of disc diffusion test. All the 12 isolates were carrying a *mecA* gene for methicillin resistance, but only 11 of the isolates were phenotypically positive for methicillin resistance. All the aminoglycoside-resistant phenotypes carried an *aacA*-*aphD* gene, and only seven isolates carried *tetK* gene (Table-4).

**Table-4:** Phenotype of antibiotic resistance and genes detected by multiplex PCR in *S. aureus* isolates recovered in the present study.

*S. aureus* isolate number	Phenotype of resistance pattern against methicillin, aminoglycoside, and tetracycline	Genes detected by multiplex PCR for methicillin, aminoglycoside, and tetracycline
373	MET, GEN, S, TET	*mecA, aacA-aphD*
415	GEN, S, TET	*mecA, aacA-aphD, tetK*
424	MET, GEN, S, TET	*mecA, aacA-aphD, tetK*
432	MET, GEN, S, TET	*mecA, aacA-aphD, tetK*
536	MET, GEN, S, TET	*mecA, aacA-aphD*
538	MET, GEN, S, TET	*mecA, aacA-aphD, tetK*
541	MET, GEN, S, TET	*mecA, aacA-aphD, tetK*
544	MET, GEN, S, TET	*mecA, aacA-aphD*
556	MET, GEN, S, TET	*mecA, aacA-aphD*
558	MET, GEN, S	*mecA*
559	MET, GEN, S, TET	*mecA, aacA-aphD, tetK*
561	MET, GEN, S, TET	*mecA, aacA-aphD, tetK*

## Discussion

Antimicrobial resistance is a global concern worldwide. The emergence of drug-resistant virulent strains of *S. aureus*, particularly MRSA is a serious problem in the treatment and control of staphylococcal infections. MRSA is a clinically significant pathogen that is resistant to a wide variety of antibiotics and is responsible for a large number of infections worldwide [8]. The accurate and rapid diagnosis of genes responsible for antibiotic resistance is extremely important in the treatment of disease and preventing the spread of infections. In the present study, we have tried to assess the multiplex PCR assay as a rapid and accurate alternative to conventional antibiotic susceptibility test such as Kirby–Bauer disc diffusion method.

MRSA has emerged as one of the serious threats to public health worldwide [14]. Due to their ­multi-resistance properties, with intrinsic resistance to all β-lactam antibiotics, there remain limited choices of antimicrobial agents to treat many life-threatening infections caused by MRSA. The emergence of these resistant strains represents a consequential response to selective pressure imposed by antimicrobial chemotherapy and once established, they are difficult to control and eradicate. The knowledge of the prevalence of MRSA and their antibiotics in any environment is necessary for selection of appropriate treatment.

In this study, the prevalence of MRSA was very high (92%) in Udaipur area. The same high prevalence of MRSA is reported from Indore (80.89%) [15]. More than 80% of MRSA was found to be resistant to ampicillin, amoxyclav, ceftriaxone, tetracycline, streptomycin, 75.75% to ciprofloxacin, 70% to norfloxacin, 66.66% to chloramphenicol and gentamicin, and 63.63% to amikacin. Amoxyclav, ceftriaxone, tetracycline, and streptomycin are the commonly used antibiotics in veterinary medicine against which also the MRSA-acquired resistance. It is also developing resistance against ciprofloxacin, gentamicin, norfloxacin, and amikacin without leaving any antibiotic for the treatment of MRSA.

Analysis from previous studies revealed a relationship between methicillin resistance and resistance to other antibiotics [16]. This study also shows that MRSA isolates were significantly less sensitive to antibiotics as compared to MSSA isolates. Many of the isolates are resistant to all the antibiotics used (41.66%). Other researchers also observed that 32% of MRSA isolates are resistant to all commonly used antistaphylococcal agents [17,18]. The observations indicate that the incidence of resistance in staphylococcal isolates varies by geographic region and the commonly used antibiotics in that region. Due to the ability of the pathogen to acquire resistance to new classes of antimicrobial agents, surveillance on antimicrobial susceptibility pattern is of at most important in understanding new and emerging resistance trends.

The gene *mecA* confers methicillin resistance. For aminoglycoside resistance, *aacA-aphD* gene coding for bifunctional enzyme conferring cross-resistance to clinically used aminoglycosides such as gentamicin, tobramycin, kanamycin, and when overexpressed, amikacin [19]. Tetracycline resistance in *S. aureus* is either based on a modification of ribosome encoded by the widely disseminated *tetM* gene or mediated by *tetK* encoded efflux pump. Of the variety of *tet* genes’ coding for efflux mechanisms, *tetK* is most often found in *S. aureus*. All the 12 isolates tested were positive for *mecA* gene (methicillin resistance) and *aacA*-*aphD* gene (aminoglycoside resistance gene). The results of the present study agree with previous results [9], who reported that all MRSA carries *mecA* gene. All the isolates carrying *aacA-aphD* gene were resistant to either gentamicin or streptomycin or both. Of the 12 isolates characterized by multiplex PCR, only seven isolates (58.33%) carry the *tetK* gene.

Similarly, researchers also developed a simple multiplex PCR assay capable of screening *S. aureus* for the presence of antiseptic resistance genes for chlorhexidine and quaternary ammonium compounds as well as mupirocin and methicillin-resistant genes, while simultaneously discriminating *S. aureus* from coagulase-negative staphylococci [8]. Our study was in accordance with a previous report [9] where we got 91.66% correlation between the phenotype and genotype of methicillin and a 100% correlation in the case of aminoglycoside, but there was only 58.33% correlation in the case of tetracycline resistance which carries *tetK* genes. The tetracycline resistance in *S. aureus* iseither based on ribosomal modification encoded by *tetM* gene or *tetK*-mediated efflux pump. As we have included only *tetK* gene in the multiple reactions, we got low correlation for tetracycline resistance and for better correlation both *tetM* and *tetK* gene should be included. This shows that the multiplex PCR reactions targeting the antibiotic-resistant genes can be a promising alternative for the conventional Kirby–Bauer disc diffusion method and broth microdilution method for the determination of antibiotic susceptibility pattern. Multiplex real-time PCR proofed to be reliable for rapid screening of high sample number and therefore could be an important tool for epidemiological purposes and support of infection control measures [20]. Multiplex molecular beacon PCR assay for detection of Enterobacteria with a broad range of β lactamases directly from perianal swabs is a sensitive and specific tool with a turnaround time of <6 h [21]. The multiplex PCR assay offers a rapid, simple, and accurate identification method for antibiotic resistance profile and could be used in clinical diagnosis as well as for the surveillance of antibiotic resistance determinants in epidemiological studies.

## Conclusion

From this study, we can conclude that there is a high correlation between phenotype and genotype of antibiotic resistance. Hence, if we are targeting all the genes responsible for antibiotic resistance, the multiplex PCR will be a better alternative to conventional antibiotic resistance sensitivity test. The detection of antibiotic resistance genes by PCR can be done within few hours, which will reduce the gap between sample submission and result.

## Authors’ Contributions

KR carried out the research work, BJ planned, designed, and supervised the experiment, DKS, AG, and SKS assisted in collection of sample and analysis and interpretation of the result, MM, PP, and LS assisted in carrying out the work, and CP participated in the review process and incorporated valuable suggestions for the improvement of manuscript. All authors read and approved the final manuscript.
